# Linkage mapping and genome-wide association study reveals conservative QTL and candidate genes for *Fusarium* rot resistance in maize

**DOI:** 10.1186/s12864-020-6733-7

**Published:** 2020-05-12

**Authors:** Yabin Wu, Zijian Zhou, Chaopei Dong, Jiafa Chen, Junqiang Ding, Xuecai Zhang, Cong Mu, Yuna Chen, Xiaopeng Li, Huimin Li, Yanan Han, Ruixia Wang, Xiaodong Sun, Jingjing Li, Xiaodong Dai, Weibin Song, Wei Chen, Jianyu Wu

**Affiliations:** 1 College of Agronomy, Henan Agricultural University, Zhengzhou, 450002 China; 2 Global Maize Program, International Maize and Wheat Improvement Center (CIMMYT), Apdo 6-641, 06600 Mexico, DF Mexico; 3 College of Life Sciences, Synergetic Innovation Center of Henan Grain Crops and National Key Laboratory of Wheat and Maize Crop Science, Henan Agricultural University, Zhengzhou, 450002 China

**Keywords:** Maize, Ear rot, Disease resistance, QTL, GWAS, Candidate gene

## Abstract

**Background:**

*Fusarium* ear rot (FER) caused by *Fusarium verticillioides* is a major disease of maize that reduces grain yield and quality globally. However, there have been few reports of major loci for FER were verified and cloned.

**Result:**

To gain a comprehensive understanding of the genetic basis of natural variation in FER resistance, a recombinant inbred lines (RIL) population and one panel of inbred lines were used to map quantitative trait loci (QTL) for resistance. As a result, a total of 10 QTL were identified by linkage mapping under four environments, which were located on six chromosomes and explained 1.0–7.1% of the phenotypic variation. Epistatic mapping detected four pairs of QTL that showed significant epistasis effects, explaining 2.1–3.0% of the phenotypic variation. Additionally, 18 single nucleotide polymorphisms (SNPs) were identified across the whole genome by genome-wide association study (GWAS) under five environments. Compared linkage and association mapping revealed five common intervals located on chromosomes 3, 4, and 5 associated with FER resistance, four of which were verified in different near-isogenic lines (NILs) populations. GWAS identified three candidate genes in these consistent intervals, which belonged to the Glutaredoxin protein family, actin-depolymerizing factors (ADFs), and AMP-binding proteins. In addition, two verified FER QTL regions were found consistent with *Fusarium* cob rot (FCR) and *Fusarium* seed rot (FSR).

**Conclusions:**

These results revealed that multi pathways were involved in FER resistance, which was a complex trait that was controlled by multiple genes with minor effects, and provided important QTL and genes, which could be used in molecular breeding for resistance.

## Background

*Fusarium* ear rot (FER) is one of the most important food and feed safety challenges in global maize production [1]. FER not only reduces the yield and quality of harvested maize but also is fatal to humans and animals, which consume the contaminated grain containing mycotoxins from some of the *Fusarium* spp. [2]. More than 10 *Fusarium* spp. can cause ear rot, among them, *Fusarium verticillioides* [synonym *F. moniliforme* Sheldon] and *F. graminearum* are the two most important species which can cause FER and Gibberella ear rot, respectively [3–5].

*Fusarium verticillioides* is an important maize pathogen in the world, which can lead to serious economic losses [6], particularly in China [7–9], the United States [10] and Southern Europe [11, 12]. *Fusarium verticillioides* can survive in plant residue, healthy seeds and soil, and initiate the infection of maize from seedborne or airborne inoculum, causing seedling disease, *Fusarium* stalk rot and FER [10, 13]. FER usually occurs on physically injured kernels, random kernels, or groups of kernels, and consists of a light pink or white mold [10]. Infected maize kernels contain toxic fumonisin that is carcinogenic in humans and livestock and even causes porcine pulmonary edema, equine leukoencephalomalacia and rat hepatocarcinoma [14, 15]. Chemical and agronomic measures are not very effective in controlling FER [1]. The best strategy to control FER and to reduce the incidence of fumonisin contamination is breeding and promoting maize varieties with genetic resistance in order [16]. Moreover, in a RIL population from NC300 × B104, Robertson et al. [17] found the genomic and phenotypic correlations between FER and fumonisin accumulation is 0.87 and 0.64, respectively, indicating that it was possible to select lines with reduced FER and fumonisin contamination at the same time [17]. These strategies require us to understand the genetics of resistance clearly, and identify the alleles that can significantly reduce the hazard from *F. verticillioides* [16].

Resistance to FER is complex because it is characterized by a quantitative inheritance in which additive, dominant, epistatic, and genotype by environment interaction effects are important [18–21]. Based on biparental populations, Mapping studies have shown that resistance to FER is controlled by many genes with relatively small effects that vary in environments and populations [4, 22]. Although different maize inbred lines and hybrids own different genetic variation for resistance to FER, there is no evidence of maize materials with complete resistance to either FER or fumonisin contamination in maize [23–25]. It is very important to identify novel resistance genes against *F. verticillioides* in order to find a lasting solution to FER problems in maize production. Several studies have identified QTL associated with resistance to *F. verticillioides* and subsequent reduced fumonisin accumulation using cross-populations, such as F2, F2:3, RILs. Zhang et al. detected six and four QTL in a F2 population of 230 individuals in two environments, respectively, and two QTL were identified consistently in both environments [26]. Using a F2:3 population, Pérez-Brito et al. [21] detected 13 QTL for kernel resistance to FER, which displayed significant QTL × environment interactions, and Chen et al. [19] discovered a QTL for FER resistance affecting approximately 18% of the phenotypic variation and accounting for up to 35% of the phenotypic effect in near isogenic lines when in homozygosity. In two additional studies based on RIL populations, Ding et al. [20] detected two QTL on chromosome 3 (bin3.04), which were consistently identified across all environments, and found significant epistatic effects among QTL and interactions effects between mapped loci and environments, and Li et al. [27] detected a resistance QTL with 10.2% of the phenotypic variation, but no epistatic effects were detected. In addition, complexity of FER could be associated with grain moisture content (GM) and European corn borer (*Ostrinia nubilalis*) [28, 29].

Recently, to uncover genomic regions associated with reduced FER and fumonisin B1 (FB1) mycotoxin contamination and identify molecular markers to perform marker-assisted selection, Maschietto et al. [30] used an F2:3 population of 188 progenies developed by crossing CO441 (resistant) and CO354 (susceptible) genotypes and evaluated FER severity and FB1 contamination content, and detected 15 QTL for FER and 17 QTL for FB1 contamination. Eight QTL located on chromosomes 1, 2, 3, 6, 7, and 9 were in common between FER and FB1, making the selection of genotypes possible with resistance to FER and low fumonisin contamination [30]. Certainly, there are many other studies on resistance to FER based on linkage mapping. This approach is widely used because linkage mapping generates lower false positive results which make up the defect of few alleles in offspring populations [31–33]. However, no genes have been isolated by map-based cloning to date, and few stable QTL have been verified for molecular breeding.

GWAS has shown enormous potential for detecting QTL with high resolution in diverse germplasm [34]. A large number of recombinational events and tens of thousands of SNPs increase the accuracy and shorten the confidence interval of QTL mapping. Now many quantitative traits have been successfully studied by GWAS in maize [35]. In 2016, Chen and his colleagues presented 45 SNPs that were significantly related to FER resistance, each of which had relatively small additive effects on FER resistance and explained 1–4% of phenotypic variation [36]. In addition, works on GWAS for FER have been performed by many other research teams, such as [37–40], and so on. Compared to traditional linkage analysis, association mapping offers higher mapping resolution and eliminates the time and cost of developing synthetic mapping populations, which make up the defect of false positive [41, 42]. So combining GWAS and linkage mapping could play a great role in identifying casual loci [43, 44].

In this study, we reused linkage mapping to identify genomic regions associated with FER resistance in a biparental RIL population that was evaluated across four environments. Then, GWAS was performed based on the data collected from five environments to detect alleles associated with resistance to FER. Next, we validated four common genomic regions in NIL populations and analyzed the candidate genes in these regions. Last, we discussed the probable mechanism of resistance to FER and stable QTL for molecular breeding.

## Results

### Phenotypic analysis

First of all, we determined the best time of inoculation for ear rot. For determining the proper inoculation time, we evaluated the phenotype of six inoculation periods of the resistant materials, BT-1 and CML295, and susceptible N6 from the 5th to the 35th day after silking (Fig. S1). The resistant materials BT-1 and CML295 showed susceptibility from the 5th to 10th day after silking, but were stable and resistant from the 15th to 35th day. The FER resistance of susceptible N6 became more and more resistant from the 5th to 35th day after silking. However, the most significant difference in resistance between N6 and BT-1 or CML295 was from the inoculation on 15th day after silking; thus, it was effortless to evaluate the materials inoculated at this time.

Descriptive statistics for FER resistance in the RIL and GWAS populations are presented in Table 1. There was a visible difference in resistance between parent lines BT-1 and N6, which had combined means 1.10 and 6.11, respectively (Fig. S2). The wide variations were also observed in each environment in the RIL and GWAS population, which ranged from 1 to 7. The frequency of phenotypic value of the GWAS population for resistance followed an approximately normal distribution, but a skewed distribution in the RIL (Fig. S3). The genotypic variance (σ^2^_g_) and genotype-by-environment variance (σ^2^_ge_) of resistance were significant in both populations. Heritability for resistance was 0.81 in the RIL population, 0.79 in the GWAS population. The high heritability indicated that much of the phenotypic variance was genetically controlled in the populations and suitable for QTL mapping.

**Table 1:** Descriptive statistics of FER resistance for the RIL and GWAS populations in different environments

Population	Environment	BT-1	N6	Mean	Range	CV	Skewness	Kurtosis	σ^2^_g_	σ^2^_ge_	*H*^*2*^
		Mean	Mean								
RIL	2007ZZ	1.02 ± 0.10	6.30 ± 0.04	1.99 ± 0.93	1–6	0.46	1.84	5.10	0.83**	–	0.90
	2008ZZ	1.26 ± 0.14	6.71 ± 0.35	2.06 ± 0.90	1–6	0.44	1.06	2.20	0.76**	–	0.88
	2015WX	1.10 ± 0.22	5.40 ± 0.18	2.23 ± 0.92	1–6	0.41	0.88	0.90	0.73**	–	0.75
	2016XC	1.30 ± 0.19	6.28 ± 0.32	2.06 ± 1.26	1–7	0.61	1.88	4.28	1.41**	–	0.73
	Combined	1.10 ± 0.17	6.11 ± 0.20	2.13 ± 0.75	1–6	0.39	1.49	3.99	0.60**	0.34**	0.81
GWAS	2014ZZ	–	–	2.87 ± 1.05	1–7	0.41	1.10	2.27	0.83**	–	0.65
	2015ZZ	–	–	3.00 ± 0.78	1–5	0.25	0.44	0.27	0.39**	–	0.65
	2015WX	–	–	2.81 ± 1.03	1–6	0.40	0.50	−0.11	0.82*	–	0.77
	2016ZZ	–	–	2.25 ± 1.14	1–6	0.55	0.97	0.26	1.08**	–	0.70
	2016XC	–	–	2.28 ± 1.17	1–7	0.57	1.48	2.77	1.04**	–	0.68
	Combined	–	–	2.75 ± 1.32	1–5	0.30	0.84	0.73	0.47**	0.33**	0.79

mean, ± standard deviation; CV, coefficient of variation; σ^2^_g_, genetic variance; σ^2^_ge_, genotype–environment interactions variance; *H*^*2*^, broad-sense heritabilities

**Significant at *P* < 0.01

### QTL mapping analysis

A total of 10 QTL were identified for FER resistance (Table 2, Fig. S4), which were located on Chr. 1 (bin 1.02/03), Chr. 2 (bin 2.00/01), Chr. 3 (bin 3.01/02, 3.06/07), Chr. 4 (bin 4.05, 4.05/06, 4.08), Chr. 5 (bin 5.00, 5.03/04), and Chr. 10 (bin 10.6/07). The increasing resistance effect of eight QTL originated from the resistant parent BT-1, whereas only two QTL from the susceptible parent N6. Among these QTL, three QTL were located on chromosome 4 (bin 4.05/08) and the one WQ6 (bin 4.05/06) between markers mmc0371 and A007339 had the largest resistance effect for *Fusarium* ear rot, which could explain more than 9.3% of the phenotypic variation. Then the QTL, on bin 3.06/07 had the second largest resistance effect explaining about 4.5%. These 10 QTL showed both additive effects (A) and additive by environment effects (AbyE), but additive effects explained 25.1% of the phenotypic variation, whereas interaction effects explained only 5.5%.

**Table 2:** Quantitative trait loci (QTL) for FER resistance identified in the RIL population using the ICIM-ADD method under MET

QTL	Chromosome bin	Flanking markers	LOD^a^	Add^b^	PVE^c^	PVE1^d^	PVE2^e^
WQ1	1.02/1.03	bnlg1007-umc1403	2.9194	0.0992	1.7159	1.6136	0.1023
WQ2	2.00/2.01	umc1419-phi96100	5.3504	−0.1329	3.0405	2.9365	0.1041
WQ3	3.01/3.02	umc2256-bnlg1144	3.3221	−0.1024	1.8653	1.745	0.1203
WQ4	3.06/3.07	bnlg197-umc1399	8.8928	−0.1633	4.5355	4.367	0.1685
WQ5	4.05	bn34-indel-17	5.5677	−0.085	2.7758	1.1562	1.6196
WQ6	4.05/4.06	mmc0371-A007339	14.8914	−0.2102	9.3098	7.127	2.1828
WQ7	4.08	dupssr28-bnlg2162	2.9395	−0.0855	1.5863	1.2133	0.373
WQ8	5.00	umc1240-umc1097	2.93	0.0782	1.6397	1.0093	0.6304
WQ9	5.03/5.04	umc2298-umc1563	4.2737	−0.1193	2.4109	2.3597	0.0512
WQ10	10.06/10.07	bnlg2190-umc1196	3.571	−0.0987	1.7937	1.6105	0.1831

^a^ Log-likelihood value was calculated by the inclusive composite interval mapping of additive gene from multi-environmental trials method

^b^ Positive value indicates the resistant gene contributed by parents N6. Negative value indicates the resistance gene from BT-1

^c^ Phenotypic variation explained by QTL

^d^ Explained phenotypic variation from additive effect

^e^ Phenotypic variation explained by interaction effect between additive gene and environment

To determine the epistatic effect, epistatic QTL mapping was performed. A total of three pairs of QTL interactions were detected by the ICIM-EPI method at an LOD value of 7, which explained 3.2, 2.4, and 2.4% of the phenotypic variation (Table S1, Fig. S5). The epistatic effect between QTL with flanking markers umc2256 and bnlg1144 and QTL with umc1791 and IDP4548 had the largest effect, and explained 3.2%. Although each QTL had the negative additive effect, the interaction effect showed a positive effect, which revolved the complexity of the resistance to FER.

### GWAS for FER

**Fig. 1:**
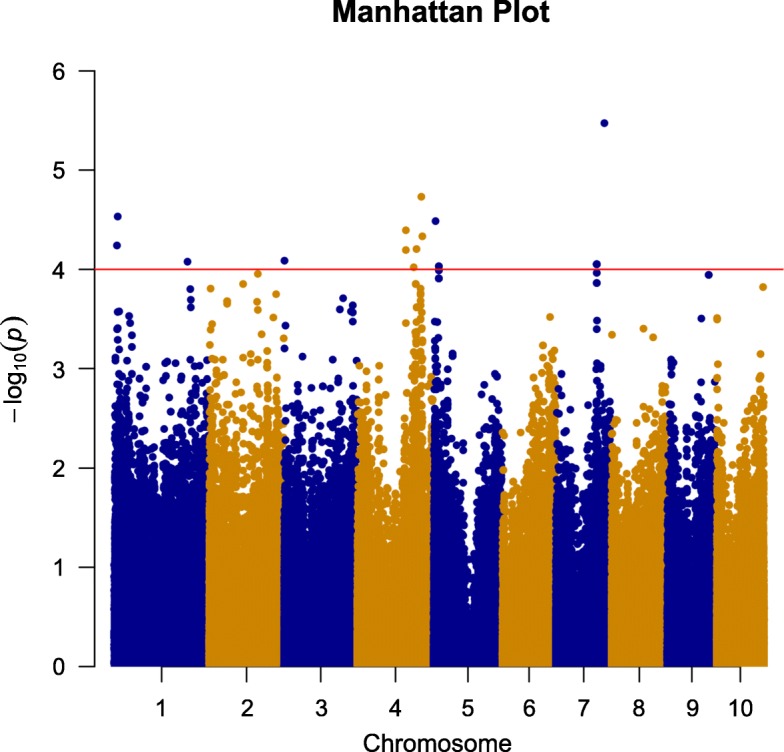
Manhattan plots of GWAS for the *F. verticillioides* ear rot resistance in maize

Single marker-based GWAS was performed using a mixed linear model (MLM) incorporating both the population structure (first three PCs) and K into the model. A total of 18 SNPs were significantly associated with FER resistance with *p* ≤ 1.0 × 10^− 4^ (Table 3, Fig. 1). These SNPs explaining 5.6 -10.2% of phenotypic variation was distributed on five chromosomes, and the number of SNPs per chromosome ranged from 1 on chromosome 3 to 6 on chromosomes 4. The most significant SNP was located on chromosome 7 (S7_153,838,246) with the lowest *P* value (3.38 × 10^− 6^) and it explained 10.2% of the phenotypic variation. The second SNP with the lowest *P* value was located on chromosome 4 and explained 6.8% of the phenotypic variation. Detailed information of 18 SNPs significantly associated with FER resistance is provided in Table 3. The QQ Plot showed that the observed *P* value was in agreement with the expected P value, whereas the observed P value was lower than the expected P value at a threshold greater than three (Fig. S6). As a result, FER may not be explained by a major gene. Some loci with lower significance may not have been detected, but this should not have affected the identification of loci significantly associated with FER resistance. Based on the physical position of the significant SNPs in the B73 version 2 reference genome, these significant SNPs were associated with 11 candidate genes, some of which were directly involved in resistance according to gene annotation, GRMZM2G150179, for example.

**Table 3:** The significant single nucleotide polymorphisms (SNPs) and their candidate genes associated with FER resistance identified in this study

SNP	Chromosome	Pos	P	R^2^	location	Candidate Gene	Annotation
S1_9,398,408	1	9,398,408	5.72E-05	0.057312	intragenic	GRMZM2G107686	Xylem serine proteinase 1
S1_11,487,039	1	11,487,039	2.93E-05	0.070112	intragenic	GRMZM2G086072	Transcription factor-like protein DPB
S1_232,529,882	1	232,529,882	8.36E-05	0.060307	intragenic	GRMZM2G150179	Putative disease resistance RPP13-like protein 1
S3_1,591,322	3	1,591,322	8.16E-05	0.056117	promoter	GRMZM2G449160	Glutaredoxin domain-containing cysteine-rich protein 1
S4_153,270,141	4	153,270,141	6.37E-05	0.058369	intragenic	GRMZM2G463471	Actin-depolymerizing factor
S4_153,270,174	4	153,270,174	4.05E-05	0.061957			
S4_178,501,587	4	178,501,587	9.53E-05	0.056872	promoter	GRMZM2G356046	Putative mannan endo-1,4-beta-mannosidase 9
S4_187,594,182	4	187,594,182	6.24E-05	0.070366	intragenic	GRMZM2G059381	chain acyl-CoA synthetase 7, peroxisomal
S4_202,889,727	4	202,889,727	1.86E-05	0.068033	–	–	–
S4_205,928,061	4	205,928,061	4.64E-05	0.062262	–	–	–
S5_6,358,869	5	6,358,869	3.27E-05	0.063575	promoter	GRMZM2G176042	Protein FAM135A
S5_16,324,316	5	16,324,316	9.26E-05	0.061203	intragenic	GRMZM2G134980	protein DnaJ
S5_16,324,318	5	16,324,318	9.26E-05	0.061203			
S7_129,966,178	7	129,966,178	8.89E-05	0.056173	promoter	GRMZM5G818643	Transcription factor bHLH49
S7_129,966,180	7	129,966,180	8.89E-05	0.056173			
S7_129,966,182	7	129,966,182	8.89E-05	0.056173			
S7_129,966,183	7	129,966,183	8.89E-05	0.056173			
S7_153,838,246	7	153,838,246	3.38E-06	0.101554	promoter	GRMZM2G488098	Unknown

Gene Ontology (GO) annotation was carried out on 11 candidate genes identified by GWAS. The process of growth, stress response, and cell formation was significantly enriched. These processes feel into four main categories, including seven associated candidate genes. The first was the cytoskeleton process, including cytoskeleton and cellular component organization, and involved candidate genes GRMZM2G449160 and GRMZM2G463471. The second was the process of protein binding, which involved the most genes, including GRMZM2G107686, GRMZM2G086072, GRMZM2G463471, GRMZM2G134980, and GRMZM5G818643, which indicated the significance in FER in posttranslational regulation. The third category was the process of regulation of cellular processes, and contained GRMZM2G107686, GRMZM2G086072, and GRMZM2G449160. The last category was the process of stress response, involving GRMZM2G059381 and GRMZM2G134980.

### Conjoint analysis for FER resistance

Ten QTL identified through linkage mapping and 18 significant single SNPs detected by GWAS were integrated to analyze the resistance, and four consistent loci were found (Table 4), located on bin3.01/3.02 (WQ4), bin4.05/4.06 (WQ5, WQ6), bin4.08 (WQ7), and bin5.00 (WQ8). These SNPs were further studied in the following experiment. From the conjoint analysis, identification of consistent loci suggested that there were resistance loci for FER with stable effects at different genetic backgrounds and environmental conditions.

**Table 4:** The consistent loci from linkage mapping and GWAS

QTL	Bin	flanking marker	SNP	position	candidate gene
WQ3	3.01/3.02	umc2256-bnlg1144	S3_1,591,322	1,591,322	GRMZM2G449160
WQ5	4.05	bn34-indel-17	S4_153,270,141	153,270,141	GRMZM2G463471
WQ6	4.05/4.06	mmc0371-A007339	S4_153,270,174	153,270,174	
WQ7	4.08	dupssr28-bnlg2162	S4_187,594,182	187,594,182	GRMZM2G059381
WQ8	5.00	umc1240-umc1097	S5_6,358,869	6,358,869	GRMZM2G176042

### QTL verification

**Fig. 2:**
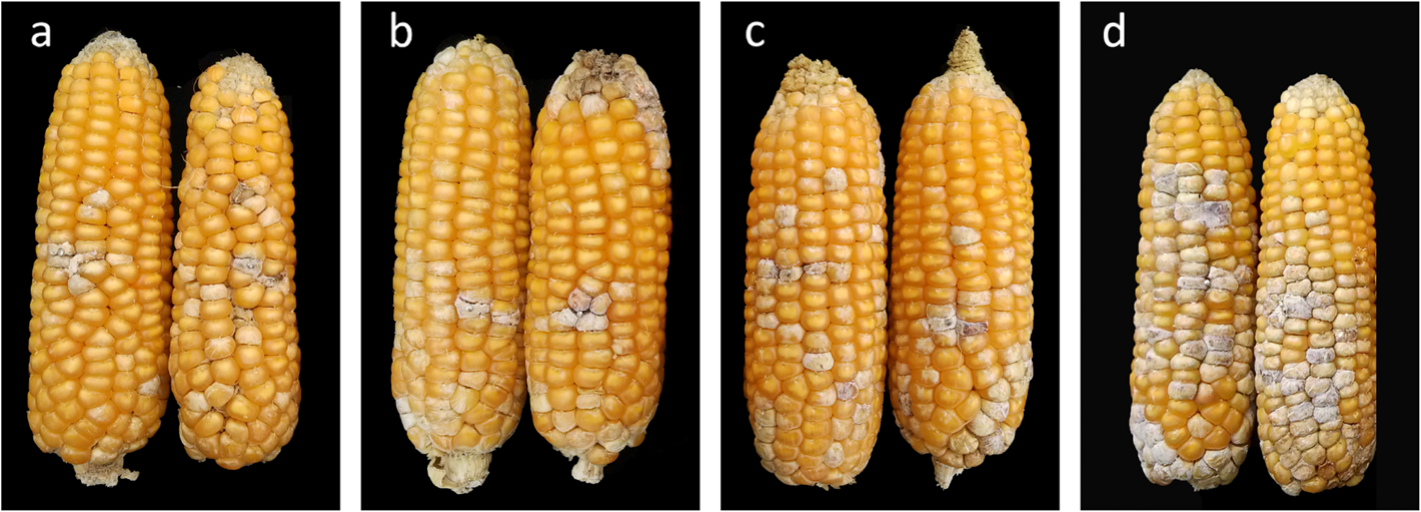
Phenotypic variation in FER severity at harvest among the NILs in artificially inoculated ears with *F. verticillioides*. N-44 is represented by the two ears on the left (**a**), N-54 (**b**), N-55 (**c**) in the middle, and N6 on the right (**d**)

To fine map the QTL (WQ5, WQ6, and WQ7) on chromosome 4, a NIL population with the genetic background of susceptible parent N6 was developed using a backcross and marker assistance selection with flanking markers. The percentage of infected kernels (PIK) was brought into the phenotype evaluation. The lines N-44 and N-54 with positive homozygous alleles (WQ5, WQ6, and WQ7) from the resistant parent BT-1 showed a lower PIK compared with N-55 and N6, and N-55 with only WQ5 and WQ6 was more resistant than parent N6, but more susceptible than lines N-44 and N-54, regardless of Zhengzhou and Xuchang. This indicated that WQ7 could decrease approximately 8 and 6 PIK. WQ5 and WQ6 together improved approximately 7 and 8% in resistance compared with N6 in Zhengzhou and Xuchang, respectively (Table 5). The analysis of variance also showed the same result, which indicated that these three QTL could increase resistance to FER. The detailed genotypes and phenotypes of the three NILs can been found in the supplementary materials (Table S2, Fig. 2).

**Table 5:** The genotype and phenotype of NILs in two environments

NILs	WQ5	WQ6	WQ7	PIK (%) and significance			
				Zhengzhou		Xuchang	
N-44	**+**	**+**	**+**	5.11 ± 0.04	c	3.48 ± 0.03	c
N-54	**+**	**+**	**+**	4.15 ± 0.03	c	2.94 ± 0.02	c
N-55	**+**	**+**	–	12.60 ± 0.06	b	8.03 ± 0.04	b
N6	**–**	**–**	**–**	19.10 ± 0.12	a	16.62 ± 0.07	a

Note:+ represents for fragment from BT-1; − stands for fragment from N6; a, b, c showed the results of Multiple measures ANOVA

A segregation population was constructed for WQ3 by a backcross between the NIL, CP-1 with the target the fragment linked with umc2101 and umc2256, and recurrent parent N6. Finally, WQ3 was verified by a family with a total of 58 plants in 2017 (Table S3).

Furthermore, the GWAS also showed a total of four significant SNPs with *p* < 1 × 10^− 4^ in these verified QTL and represented three candidate genes: GRMZM2G449160 for WQ3, GRMZM2G463471 for WQ5 and WQ6, and GRMZM2G059381 for WQ7 (Table 4).

## Discussion

### QTL analysis and GWAS for FER resistance

QTL analysis is a well-established and widely-used tool for dissecting the genetic basis of complex traits in plants [45]. Many QTL associated with important agronomic traits have been mapped but only a few causal genes were cloned in cereals [46, 47]. Similarly, to date, many QTL have been mapped, but no causal genes have been cloned underlying QTL for FER resistance controlled by many minor-effect QTL that play a great role in maize [48]. These indicate that the positional cloning of minor-effect QTL is still difficult because of their low heritability. Compared to traditional linkage-based analyses, GWAS offers higher mapping effects containing mapping resolution and a greater number of loci, because of many polymorphic SNPs, and eliminates the time and cost associated with developing synthetic mapping populations [41, 42]. However, GWAS easily generates false positive results because of the population structure. Thus, combining GWAS and linkage mapping could exploit the complementary strengths of both approaches to identify casual loci or genes [43, 44].

To decrease the loss from FER and explore the genetic mechanism, we begin to study resistance to FER more than a decade years ago. Today, we have formed a series of relatively perfect inoculating systems and phenotypic identification methods [49], and have achieved some degree of success [19, 27, 36, 50–52]. In this study, 10 QTL and 18 SNPs (*P* < 1 × 10^− 4^) were detected on the whole genome. Among them, four significant SNPs were located in four QTL, which represented three candidate genes, GRMZM2G449160, GRMZM2G463471, and GRMZM2G059381. GRMZM2G449160 is a member of glutaredoxins (GRXs), which belongs to the antioxidants involved in cellular stress responses. Proteomic analysis found that homologous *OsGRX20* increased by 2.7-fold after infection by bacterial blight in rice [53]. GRMZM2G059381 belongs to the AMP-binding protein and the homologous *OsBIABP1* is involved in the regulation of the defense response through salicylic acid (SA) and/or jasmonic acid (JA) / ethylene (ET) signaling pathways [54]. GRMZM2G463471 is a member of the actin-depolymerizing factors (ADFs), whose primarily function is to regulate the severing and depolymerization of actin filaments. However, in recent years, the activity of ADFs proteins has been linked to a variety of cellular processes, including those associated with responses to stress. Zhang et al. [55] found a member of ADFs, e.g., *TaADF4*, from wheat, was required for resistance to the stripe rust pathogen *Puccinia striiformis* f*.* sp*. Tritici*. These results indicate that the three candidate genes in this study may be associated with FER resistance in maize, which will be focused on in the following study.

### Phenotypic evaluation for FER resistance

An accurate phenotype is the key to the study of FER. The acquisition of the phenotype was influenced by the inoculation method, date, and the inoculation dose. At present, there are three common inoculation methods used for the study of FER resistance, namely the silk channel inoculation method [56, 57], silk sprayed with inoculum method [4], and the sponge and nail punch method [58]. Among them, the last method is widely used because of easy control of the inoculation dose.

In the long-term study of FER, we explored and optimized the nail punch method [49]. The key to this approach is the operation timing of inoculation. This method is most suitable for inoculation in the milk ripening period, the 15th day after silking, because earlier or later inoculation can not accurately reflect the resistance of the materials. The most significant difference in resistance between susceptible inbred line N6 and resistant inbred line BT-1 or CML295 was from the inoculation on 15th day after silking; thus, it was effortless to evaluate the materials inoculated at this time. To assess the resistance of polymorphic GWAS population, it was divided into two parts according to the date after silking and planted at two different times to ensure the same time of inoculation.

### Stable QTL for *Fusarium* resistance in different tissues and studies

**Fig. 3:**
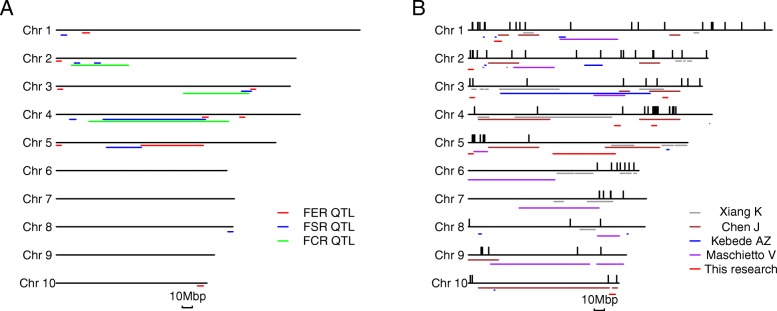
QTL and SNPs for *Fusarium* resistance in different tissues and studies. **a**) Comparison of QTL for *Fusarium* rot resistance detected by different tissues. **b**) Comparison of QTL and SNPs for *Fusarium* ear rot resistance detected by previous reports. Colored lines represent different QTL or SNPs in different studies

For more than 10 years, our group studied *Fusarium* resistance in different maize tissues [19, 27, 36, 50–52]. We confirmed that the resistance loci and mechanism of different tissues were different. In the GWAS population, we found some lines showed different resistance between different tissues, for example some lines had high FER resistance with weak *Fusarium* cob rot resistance (FCR) and *Fusarium* seed rot resistance (FSR). Therefore, we compared the QTL identified for *Fusarium* resistance in ear, cob (FCR), and seed (FSR) (Fig. 3a). These studies used the same resistance parent line and similar susceptible lines, but had different results [50, 52]. Some QTL were independent, for example, the QTL located on bin 3.01/02, bin 5.00, and bin 10.06/07 were specific for FER. However, there were still some QTL that appeared conservative; for example, QTL in bin 3.06/3.07 and 4.05/4.06 were identified in FER, FCR, and FSR. These conservative QTL were not identified for *Fusarium* seeding rot, which may cause by different resistant mechanism or different research populations [59]. The candidate genes identified by GWAS were different between these three traits, as well [50, 52, 60]. It is worth mentioning that many candidate genes for FCR were involved in cell and tissue development, whereas none of this kind of gene was identified in FER. These results suggested that the resistance against *Fusarium* in maize is a complex trait, which requires continued research to resolve this important production problem.

In our previous study [27], four QTL located in bin3.01/4.06/5.01/6.04 were detected using composite interval mapping method across two environments (2007ZZ and 2008ZZ). Among these QTL, two QTL in bin3.01/5.01 were also detected in this study, but the QTL in bin4.06 in the previous study was not detected; rather bin4.05/4.06 and bin4.08 in this study were verified by NILs. Moreover, no significant loci were detected on chromosome 6 in the RIL population and GWAS population in this analysis.

Previous studies [19, 30, 36, 37, 61, 62] together with the present study were compared, which showed that loci for FER resistance are widely distributed, indicating FER resistance is a complex trait controlled by many minor QTL (Fig. 3b). The five common loci detected in RIL and GWAS populations were also identified by other researchers showing that these QTL were stable. These results also indicated that maybe it was effective to increase resistance according to selecting lines with a few stable QTL. Furthermore, the QTL verified by NILs on bin 3.06/07 and 4.05/06 will be the focus on our following study plan, because they were identified by different research and *Fusarium* resistance traits.

## Conclusions

In this study, a RIL populations and one GWAS population were used to identify and map QTL for FER resistance. A total of 10 QTL for FER resistance were detected by QTL mapping and 18 SNPs were identified by GWAS at *P* < 1 × 10^− 4^. Four QTL of the five common loci in the RIL and GWAS were verified in NILs and three candidate genes may be associated with FER resistance. These results confirmed that FER resistance was strongly controlled by multiple genes with low effect and the QTL and candidate genes identified in this study could help to better understand the genetic basis and explore the mechanism of FER resistance. At the same time, it was feasible to select maize lines with higher *Fusarium* resistance because of the two stable QTL in different tissues and studies.

## Methods

### Germplasm and experimental design

A biparental population composed of 250 recombinant inbred lines (RILs) was constructed by a cross between inbred lines BT-1 and N6 (Fig. S2) by the single-seed descent method. The line BT-1 was reformed by tropical Asia material with highest resistance to FER, which was screened out of 90 inbred lines inoculated with *Fusarium verticillioides* [63], whereas the susceptible N6 was a Tangsipingtou line. The RIL population together with the two parents were grown in Zhengzhou (34°52′N 113°37′E) in 2007 and 2008 (2007ZZ, 2008ZZ), Wenxian (34°57′N 113°2′E) in 2015 (2015WX), and Xuchang (34°3′N 113°41′E) in 2016 (2016XC). These three areas have a temperate continental climate, with adequate rainfall and enough light-temperature resource in the growing period of maize. NILs were developed by crossing a recipient line N6 with the donor line BT-1 through five cycles of advanced backcrosses. These NILs were evaluated in 2016 and 2017.

The GWAS population consisting of 265 maize inbred lines from the heterotic populations Tangsipingtou, Reid, Lancaster, P group, and some tropical lines from the International Maize and Wheat Improve Center (CIMMYT), was evaluated in Zhengzhou in 2014, 2015, and 2016 (2014ZZ, 2015ZZ, and 2016ZZ) and Wenxian in 2015 (2015WX) and Xuchang in 2016 (2016XC).

All the populations above were laid out in a randomized complete block design with two replications in each environment. Sixteen plants were planted in 4 m row plots with 0.67 m row spacing. Importantly, pesticide was artificially sprayed at the ten-leaf stage to control corn borers. Some other field management was performed according to the standard agronomic practices in each location.

### Inoculation and FER evaluation

A single-spore isolate of *F. verticillioides* was reproduced artificially on sterile mature maize kernels, incubated for 7 d at 28 °C. Then, the spores were harvested, and the concentration was estimated using a hemocytometer and adjusted to 1 × 10^6^ spores ml^− 1^ in sterile distilled water with 0.2 μl/ml Tween 80 surfactant. The top ear of each plant in each row was inoculated for 2 ml spore suspension on the fifteenth day after silking using the sponge and the nail punch method [58] along with the portable inoculating tool assembled by ourselves.

Resistance to FER was assessed by disease severity according to Reid et al. [64] using a rating scale from 1 to 7 [64]. Ten ears of each line in a row were selected for phenotypic evaluation and the final data for each replication was the average value. The average rating scale from the two replications represented the final value in each environment.

### Phenotypic data analysis

The analysis of variance (ANOVA) of phenotype data was carried out using the multifunctional IciMapping version 4.2 [65]. Best linear unbiased predictions (BLUPs) of the combined resistance to FER for each line in Linkage analysis and GWAS were calculated by using a mixed linear model (lmer) in the R version 3.6.0 with the R stats package [66], in which used replication, environments (year-location combinations), and entries were considered random effects. The BLUP value of each line was used for GWAS analysis.

### Linkage mapping

In our previous study, we constructed a linkage map of the RIL population (BT × N6), which contained 207 polymorphic SSR markers and had a length of 1820.8 cM with an average 11.7 cM distance [27]. In this study, we reconstructed a linkage map with 222 polymorphic markers using the IciMapping version 4.2, which had a total length of 1890.7 cM and an average genetic distance of 8.5 cM between markers.

To understand the QTL by environment interaction effects, the mapping strategy of MET (mapping of additive and digenic epistasis genes from multi-environmental trials) was performed in the IciMapping software. Two methods were used in QTL mapping, i.e., (i) ICIM-ADD: Inclusive Composite Interval Mapping of additive QTL (ii) ICIM-EPI: Inclusive Composite Interval Mapping of digenic epistatic QTL. The threshold value of LOD was 2.5 for ICIM-ADD and 7 for ICIM-EPI.

### GWAS and candidate gene annotation

Through the genotyping-by-sequencing (GBS) method conducted in Cornell University, a total of 955,650 SNPs were identified in the GWAS population [67, 68], and the SNP flanking sequence and position information were available on the “panzea” website (http://cbsusrv04.tc.cornell.edu/users/panzea/download.aspx?filegroupid=4). The filtered parameters of SNPs, linkage disequilibrium (LD) between each pair of SNPs, the principal component analysis (PCA), Kinship matrix and population structure analysis were performed according to our previous study [69].

Candidate gene information was obtained from the MaizeGDB (http://www.maizegdb.org/) genome browser based on the physical position of significant SNPs in B73 RefGen_v2.

## Supplementary information

**Additional file 1:**

**Additional file 2:**

## Data Availability

The datasets used and/or analysed during the current study are available from the corresponding author on reasonable request.

## Abbreviations

FER: *Fusarium* ear rot
RIL: Recombinant inbred lines
QTL: Quantitative trait loci
SNPs: Single nucleotide polymorphisms
GWAS: Genome-wide association study
NILs: Near-isogenic lines
ADFs: Actin-depolymerizing factors
FCR: *Fusarium* cob rot
FSR: *Fusarium* seed rot
GO: Gene Ontology
PIK: Percentage of infected kernels
