# Supplementary material for: Linkage mapping and genome-wide association study reveals conservative QTL and candidate genes for Fusarium rot resistance in maize
Source: BMC Genomics. 2020 May 12;21:357. doi: 10.1186/s12864-020-6733-7 (PMC7218626; doi:10.1186/s12864-020-6733-7)
Supplement: Supplementary file 1 — Additional file 1: [file 12864_2020_6733_MOESM1_ESM.pptx]

## Slide 1
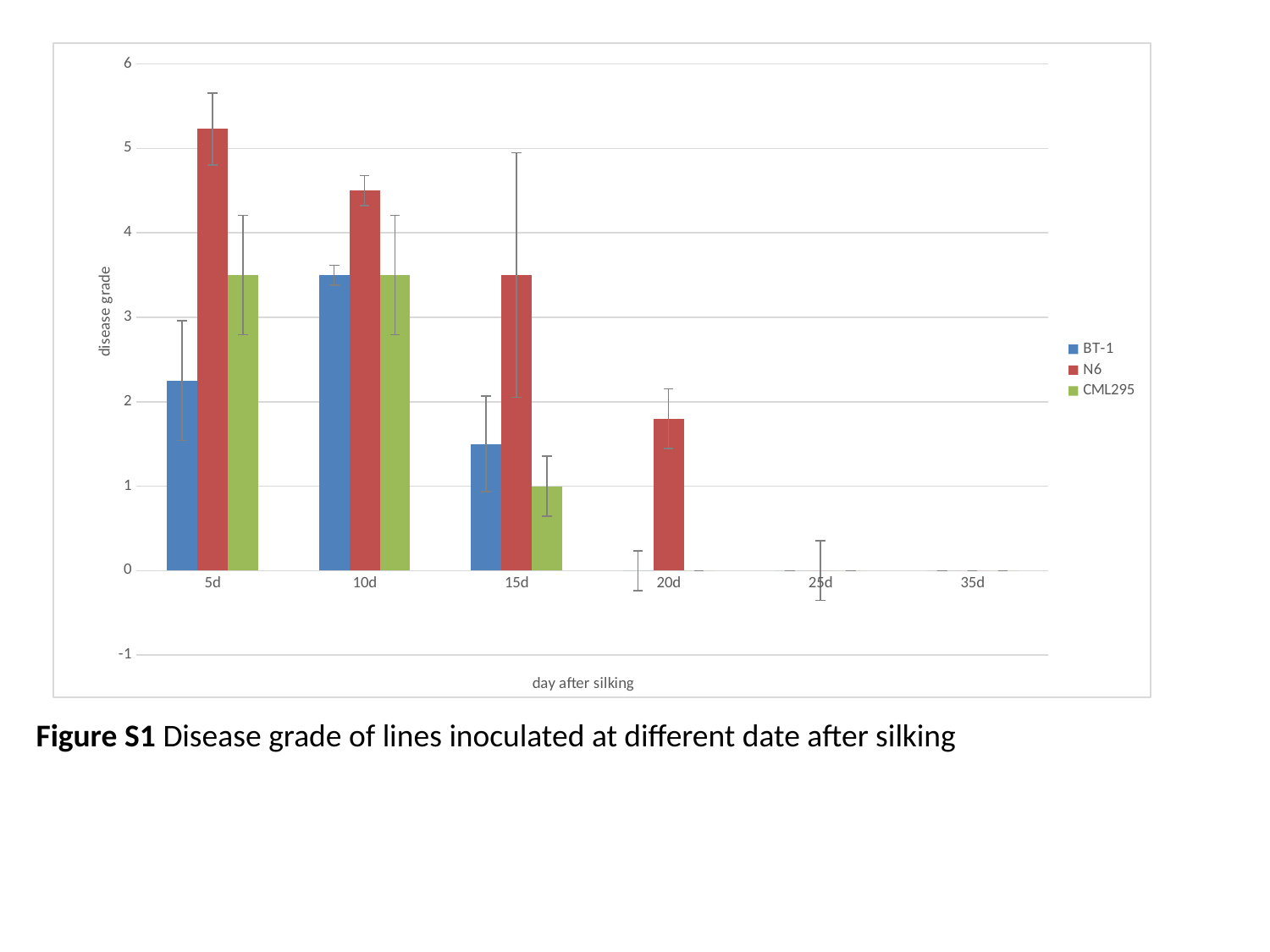

### Chart
| Category | BT-1 | N6 | CML295 |
|---|---|---|---|
| 5d | 2.25 | 5.23 | 3.5 |
| 10d | 3.5 | 4.5 | 3.5 |
| 15d | 1.5 | 3.5 | 1.0 |
| 20d | 0.0 | 1.8 | 0.0 |
| 25d | 0.0 | 0.0 | 0.0 |
| 35d | 0.0 | 0.0 | 0.0 |Figure S1 Disease grade of lines inoculated at different date after silking

## Slide 2
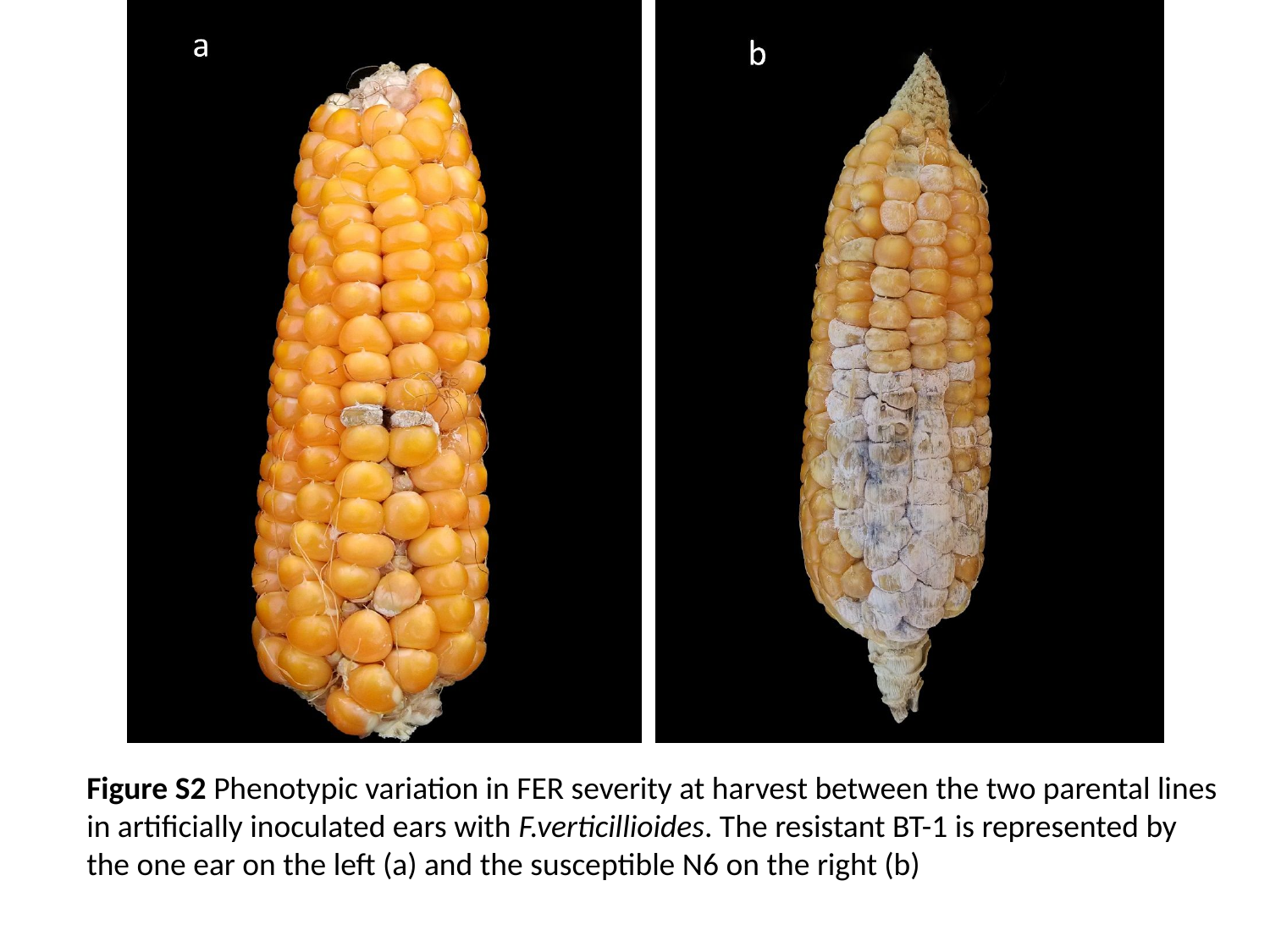

Figure S2 Phenotypic variation in FER severity at harvest between the two parental lines in artificially inoculated ears with F.verticillioides. The resistant BT-1 is represented by the one ear on the left (a) and the susceptible N6 on the right (b)

## Slide 3
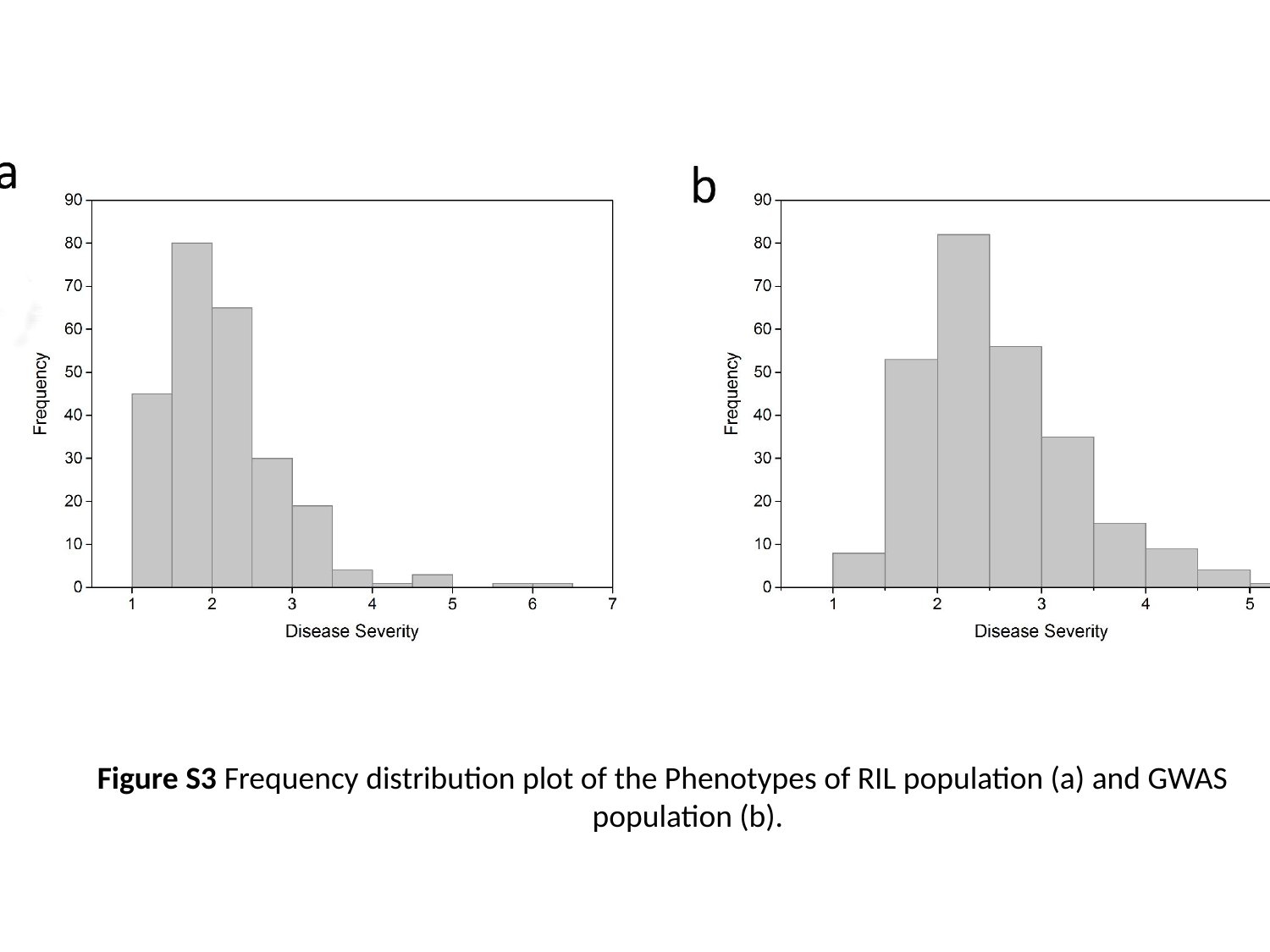

Figure S3 Frequency distribution plot of the Phenotypes of RIL population (a) and GWAS population (b).

## Slide 4
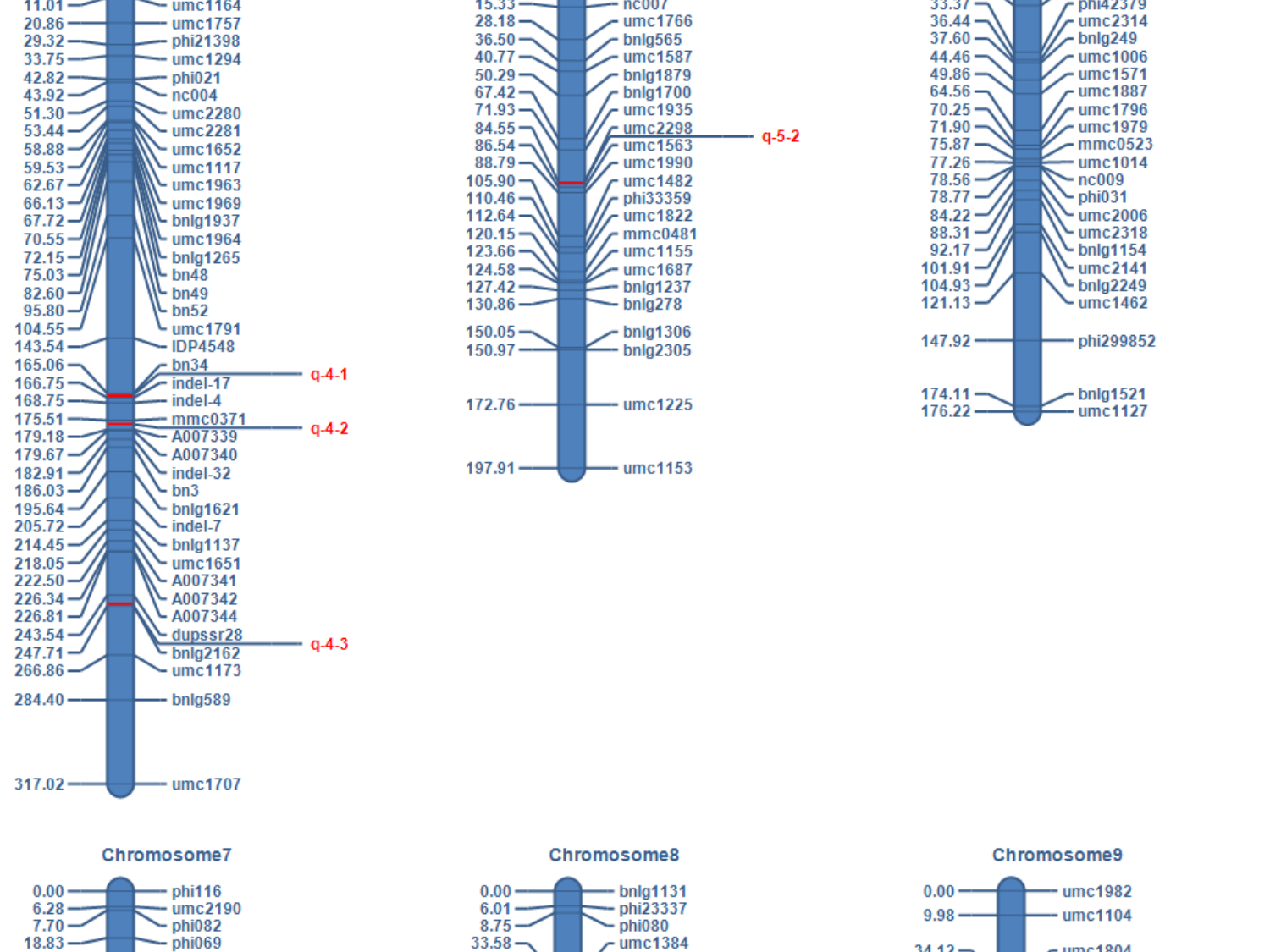

Figure S4 Linkage map consisting of 222 markers and QTL detected from the whole genome in RIL population.

## Slide 5
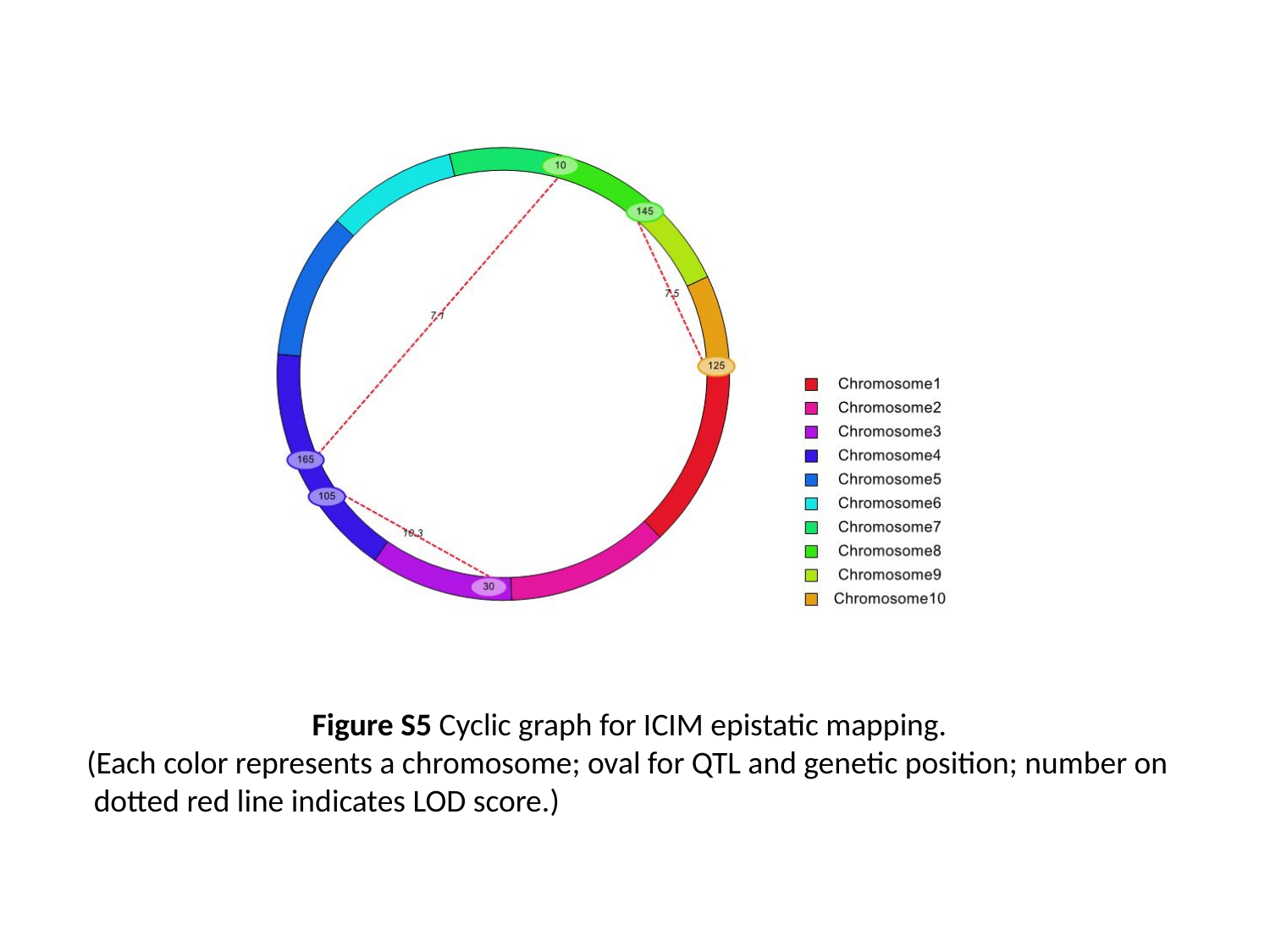

Figure S5 Cyclic graph for ICIM epistatic mapping.
(Each color represents a chromosome; oval for QTL and genetic position; number on dotted red line indicates LOD score.)

## Slide 6
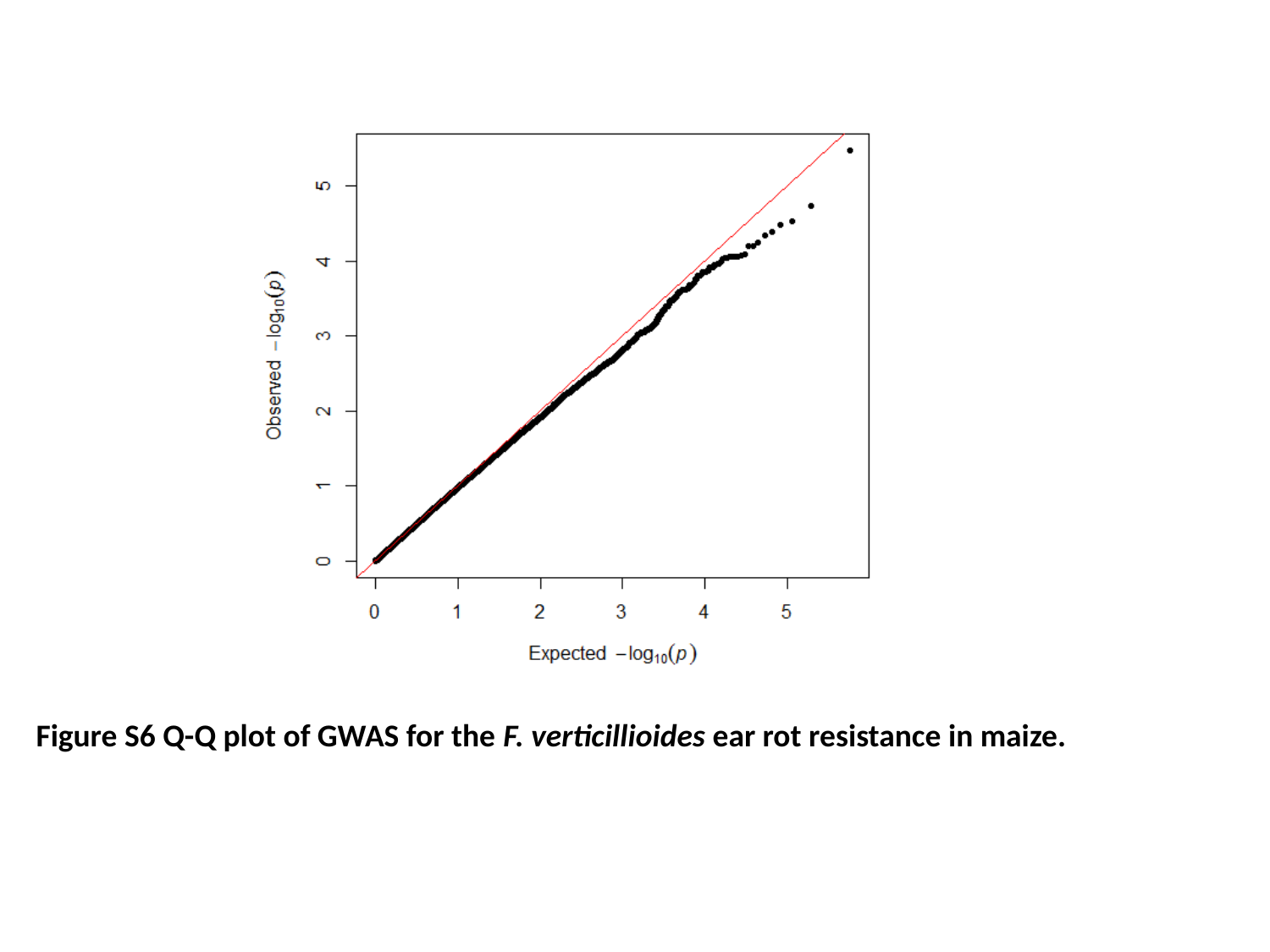

Figure S6 Q-Q plot of GWAS for the F. verticillioides ear rot resistance in maize.
